# Notes on Medical Matters and Medical Men in Paris

**Published:** 1848-02

**Authors:** David W. Yandell

**Affiliations:** Louisville, Ky.; Paris


### WESTERN JOURNAL

OF

### MEDICINE AND SURGERY.

FEBRUARY, 1848.

### Art. I.—*Notes on Medical Matters and Medical Men in Paris.*—By David W. Yandell, M.D., of Louisville, Ky.

Having spent nearly four months in a tour through Europe, in which I have seen the principal cities of the Continent, I find myself at the beginning of the lecture term once more in beautiful Paris. At some future day, when I have more leisure, I may give you some account of the things medical seen on my journey—of the noted medical men of Berlin, Vienna, Venice, Florence, and Rome—and of the medical institutions of Germany and Italy; but at present, amid the pressing calls upon my time made by studies which must not be neglected, I can do nothing more than send you a few of the practical details gathered in my attendance at the hospitals.

*Velpeau on Fractures of the Femur.*—M. Velpeau, in his last annual summary of the cases that had been treated in his wards during the scholastic year, 1846–'47, among other things, spoke as follows of Fractures of the Femur:

You have had opportunities for observing twelve cases of fracture of the femur, among which were fractures of the body, of the neck, and of the extremity or condyles of this bone. We have had only one example of this latter variety; this was in an exceedingly intractable little boy, who, in a fall had broken the external condyle very obliquely. This single case, however, has sufficed to show you the difficulty of precise diagnosis, as well as of the coaptation of the parts, and how important it is not to confound this variety with fracture of the body, the treatment and prognosis of which are essentially different.

The danger of fractures of the inferior extremity of the femur are of two kinds, and relate—1st, to the possibility of inflammation of the knee; 2d, to the production of a more or less considerable deformity. This last accident may itself be grave in two ways; the joint, deformed either by the fact of the fracture alone, or by the irregularity of the consolidation, will present inequalities that will produce stiffness in the movements, perhaps a demi-anchylosis; or, the extremity of the femur, badly consolidated, as nearly always happens, will offer projections which, pressing on the surrounding tissues, will give rise to a more or less severe and more or less constant pain, and lead to ulceration of the vessels, nerves, skin, etc. As I have hinted, the treatment of fractures of the condyles and of fractures of the body of the femur is entirely different, the former offering difficulties that you do not encounter in the latter case. This is owing to the fact that fractures of the condyles are ordinarily unequal, irregular, angular; and, as in this region the bones are naturally irregular, covered with projections or points, it is very difficult to distinguish, in the midst of the tumefaction of the soft parts, what are the connections, extent, and form of the fragments. These difficulties which you experience in establishing a precise diagnosis imply that there are no fixed rules of treatment; for how can you deal with fragments of which you know neither the form nor direction? How effect an exact coaptation? Especially, how can you maintain these fragments on which you have no hold? Thus it is we are obliged to grope our way; here you have the reasons which induce us to place by turns the leg in the various degrees of flexion, extension, and rotation, until we succeed in bringing the fragments in a favorable position. But even here, the muscular force acts against the efforts of the surgeon; the muscles in drawing upon the leg tend to elevate the tibia, and place it upon the fragments, which it slips between, separates, and produces a transverse displacement of greater or less extent.

You were able to observe all these accidents in the case of our little patient whose indocility added to and increased the difficulties just enumerated. You saw the irritation caused by the irregularity of the fragments produce an abscess, which gave me for an instant much uneasiness for the joint; though this abscess, once opened, improved very rapidly and the patient left the hospital cured. You have had an opportunity of seeing him since, as he returned and passed some days in our ward for a light contusion situated on the right leg.

It has been your good fortune to have had opportunities in the course of this year for observing the three varieties of fracture of the neck of the femur. We have had, you will remember, patients in whom the fracture was seated within the articular capsule, (*intra capsular fracture*); in others the solution of continuity was external to the capsule, (*extra-capsular fracture*); finally, some have offered the modification called *fracture by penetration*, in which the neck of the femur was *buried* in the head of the bone. Two patients have presented us the characters of this last species of fracture; that is, with them, there was notable shortening of the limb, rotation of the foot outwards, absence of crepitation, and the leg could be lifted up. If the member did not lose its functions, if there was not immobility, it was owing to the fact that the neck having entered the head of the femur, the penetrated fragment maintained the fragment penetrating it.

In the extra-capsular fractures, there is projection at the hip; the pain is seated beneath the articulation; there is shortening, mobility; possibility of giving to the member its length; the leg cannot be raised; the point of the foot is turned outwards.

In intra-capsular fracture, the fold of the groin is effaced; the pain exists on a level with the articulation; the rotation outwards is more marked than in the former case; shortening; loss of functions of the member.

You have been able to see that if these fractures have a certain gravity, it is much more by reason of the advanced age of the patients, of the necessity of confining them to bed, than to the solution of continuity itself. There are no important organs or viscera in this neighborhood to be wounded. This class of fractures occurs mostly in old people, and one is hardly at a loss to comprehend that a casualty which requires two or three months of confinement, is serious. Nevertheless, it should be remarked that certain old persons support the immobility without any great inconvenience.

You may remember the woman, 83 years old, who kept her bed for more than two months without accident, without her health appearing affected. But I must insist that the contrary is most often the case, and one can easily conceive that there are very few healthy persons who after keeping their beds for a certain length of time, would fail to become diseased, for exercise and motion are necessary to the integrity of the functions; the circulation languishes, the digestive organs grow sluggish, no longer act with their accustomed energy, and finally become covered with a muddy coat, which alters the mucous membrane. Nothing is more certain than that if you place an old person in these conditions he runs the greatest risk; he loses his appetite, then comes diarrhea, marasmus, and often death. I hope you will remember this; many old persons who are treated for fracture of the neck of the femur die in this way.

Another danger attendant upon fracture of the neck is owing to the prolonged compression experienced by certain projecting regions; such as the eschars of the sacrum, grand trochanter, etc.; eschars which may penetrate to the bones and change them; which produce an abundant suppuration, and react upon the whole economy in an unpleasant manner. It is in virtue of these various circumstances that the fractures of the neck of the femur possess gravity.

The question of the consolidation of intra-capsular fractures has been differently resolved. Sir Astley Cooper rejected the possibility of consolidation, because the head of the bone, receiving fewer vessels, no longer preserved the same vital energy, and could not furnish the elements of the reparatory work. This opinion, however strange it may appear at first glance, found numerous partisans, among whom was a celebrated surgeon of Montpellier, Delpech, who went so far as to offer a prize of two thousand francs to the person who would show him a consolidated fracture of the neck of the femur. It is also true that he afterwards awarded the prize to himself.

One of the first who examined the other side of the question was Smith, who published a memoir accompanied by plates, and endeavored to prove that these fractures could be consolidated. But it is apparent from the difficulty which he had in collecting examples that this consolidation, if it is possible, is also extremely rare. It is clear, that in the observations he cites, the consolidation is real; but these facts appear to belong to the category of fractures by penetration. Now, for my own part, I no longer consider the consolidation of fractures of this kind to be an open question. It was not of those that Sir Astley Cooper meant to speak, because they had attracted very little attention up to that time. The memoir of Smith, then, does not demonstrate the possibility of the consolidation of intra-capsular fractures, without penetration of the fragments.

Even in the mass of facts collected by M. Chassaignac, and borrowed especially from Amesbury, Stanley, Vanhonte, Brulatour, etc., one sees that if these fractures do consolidate, such instances must be rare; and that in no case is the consolidation ever complete as in the body of the bone.

How can we comprehend, in fact, that intra-capsular fracture can be completely consolidated, when we remember that the head is no longer held by any thing but the round ligament, which contains only a few capillary vessels? How hope, under such circumstances, that a complete callus will be developed, especially when it is impossible to maintain the two fragments in perfect coaptation? A priori, then, the consolidation appears difficult; and since the facts collected and the observations published, are not of a nature to remove all doubt, one is right in still doubting.

Here, now, is probably what passes: there is not a complete callus, but a considerable work is established, which leads to the formation, within the capsule, of osseous plates—of stalactites. These osseous plates, at first small, gradually extend, join to one another, and form an imperfect mass which encases the neck of the bone, re-establishes its solidity, and thus causes the thigh to recover its movements. But in certain cases, the capsule becomes itself encrusted with calcareous matter, the movements remain, embarrassed, difficult, and the lameness persists.

From all this, one may deduce a therapeutic consequence. Why treat these fractures, since, as I have shown you, the treatment may be attended with serious inconveniences and the consolidation would be irregular?

This is the starting point of the doctrine of Cooper.

I will not speak of the various bandages invented for the treatment of fractures of the neck of the femur. For twenty years I have in some degree left the patients to themselves. If the fracture is extra-capsular, mobile, in an old person, I place the member in demi-flexion, put a cushion under the popliteal region; then at the end of fifteen or twenty days, I make the patient get up and walk with crutches. The weight of the member makes extension in the walk; the movements do not hinder the formation of stalactites, of the osseous vegetations which establish the consolidation. From this mode of treatment it results that the patients are able to walk about at the end of the third week, first with crutches, then with a cane; whereby, consequently, they do not incur all the dangers attached to a long confinement in bed; the movements of the limb are established in part, and sometimes the lameness at length disappears. At the end of six weeks upon this plan the patients are at the same point as after three, four, and five months of treatment by immobility, and they have not been exposed to the same dangers. Further, they have not had to support the pains caused by a bandage keeping up continued extension, nor all the accidents that it induces, eschars, œdema, etc. Certain surgeons reject this treatment from fear of shortening; but this is entirely chimerical; for the patient does not rest upon the weak member, he walks upon the other foot, and, as I have just now told you, the weight of the member exercises a continued extension which one must court. The fragments are not agitated by the movements, for these do not go on at the articulation of the hip where they would produce a lively pain. Of the twelve patients whom we have treated this year, one alone has died, and that occurred because we were not able make him get up.

*Cyst of the Liver.—Repeated punctures.—Consecutive Inflammation.—Influence of the air in accidental cavities.*—I propose calling your attention this morning, said M. Velpeau, to the patient, the history of whose disease since his entry into the hospital, which was many months ago, until this moment, when I intend performing a sort of operation upon him, offers us useful instruction in a practical point of view.

This man has in the right hypochondriac region a tumor belonging to the liver; I believe that this tumor was primarily hydatic, though I could in no wise detect that particular *frémissement* which has been called *bruit hydatique.*

The first time that I punctured it a transparent, citrine, limpid liquid came away; the patient was relieved, and it was sometime before the tumor filled anew. I then practiced puncture for the second time, when the liquid had another aspect; it was reddish; the tumor now refilled itself with greater promptitude. A third puncture was made which gave issue to a liquid again modified in its aspect; it was redder and thicker. A fourth puncture became necessary. It is now some weeks that well characterized pus has come away from the tumor. You see this was a cyst, at first almost inert; in consequence of the first puncture there was an irritation established which was not very intense; after the second it was more marked, and after the third, the irritation was sufficient to determine locally a very violent pain, and an inflammatory action which gave rise to a general febrile reaction to such a point, that pus was formed in the tumor and the fourth puncture gave issue to it.

Thus, this cyst, at first perfectly inactive, gradually became irritated and inflamed, and has become something more serious than it was previously. What is the cause of this change? It is most certainly not the introduction of air that we can accuse; the canula was extremely small, and all necessary precautions were taken in introducing it to prevent the introduction of air into the cavity. I repeat, that puncture practiced in the deep-seated tissues may often give rise to inflammation without the air having penetrated, without the air having any hand in it.

There are persons who think that subcutaneous punctures cannot produce inflammation; this is an error. These punc tures are not always unattended by danger; they are not as innocent as they are represented to be.

Our patient had a cyst for many years; it gave him almost no trouble; all the functions of his system were well executed; he was in almost perfect health, and now behold him in an entirely different state. Before, it was possible to leave him in the state in which we found him; now, it is impossible to leave him without treatment.

For a long time I have hesitated about treating him; for the means which we possess against his disease are not to be depended on, and may even be of dangerous tendency.

In surgery, we often have cases comparable to this, as, for example, tumors of the ovaries, fibrous bodies, which are, in a great number of circumstances, compatible with long life. For these tumors, persons have dared to open the abdomen and thus extirpate them; and it is especially in England and America, that these rash endeavors have been made. As for me, I blame them very severely; these are unreasonable attempts which are unworthy of surgery. In the beginning, I recoiled before an operation, and I had engaged our patient to quit the hospital, but he afterwards refused; he wished that I should cure him. I decided with pain to make a second puncture, and this little operation has determined an inflammation which may result fatally if I leave the patient without treatment. For thirty years his affection has been beyond the resources of art; to-day it may be combatted. A first procedure consists in incising the walls of the abdomen upon the tumor, to lay it open to its bottom and put in a pledget of lint. This is the most prompt, but also the most dangerous procedure, for the peritoneum is opened, and the matter may escape into its cavity; a double chance of peritonitis. Unless one be assured that the peritoneum is adherent, he must reject this operation. Dr. Graves, of Dublin, acts in another way. He incises the abdominal walls layer by layer; then, arrived at a few lines distance from the peritoneum, if there be adhesion, he punctures on the spot, if not, he stops and waits, hoping that a local inflammation will cause the peritoneum to adhere to the tumor and the walls of the abdomen.

This procedure is not sure; first, because it is impossible to go with certainty just to the peritoneum, without wounding or opening it, and afterwards because the inflammation, instead of being adhesive, may be purulent, and the pus, if it escapes into the abdominal cavity, will produce a peritonitis.

Further, the tissues in cicatrizing, thickening, may prevent the tumor from opening externally.

M. Begin does better. He incises in the same way to the peritoneum; then he incises the peritoneum as respects the tumor. This last naturally becoming engaged in this species of button-hole, contracts adhesions upon its sides; later, the tumor is punctured. This procedure has, to my knowledge, been employed two or three times by M. Begin; I have myself used it twice with success; but it is only applicable when the tumor is globular; if it be flat as this is, it does not engage itself in the button-hole, and its gliding upon the borders of the wound may produce an inflammatory irritation.

A fourth procedure is that of M. Récamier, and it is this that I am going to adopt in the case of our patient, because it is the best. It consists in the application of caustics to that portion of the abdomen corresponding to the tumor. It is necessary to have a deep eschar and as large as a five franc piece. Three or four grains of caustic must be applied. Then wait for or endeavor to hasten the fall of the eschar. Apply the caustic four or five times, until you have destroyed the entire thickness of the abdominal wall. You thus forcibly produce an eliminatory inflammation without, and an adhesive one within. When you arrive at the cyst, introduce the instrument. These means are then, so to speak, but preparatory. They have for their end to bring the tumor to the exterior. This obtained, you act upon this tumor as you would act upon an external one. As far as the operation goes nothing is more simple; but is this all? We have here parietes of a thickness more or less great, but slightly retractile; the tumor may always remain open, as nothing tends to obstruct the cavity.

There remains a large cavern which may secrete a pathological liquid; and as it is almost impossible to make the opening in the most depending portion, the liquid stagnates and is decomposed by the air; this air is not at all an excitant, as has been said, but merely a chemical menstruum necessary by virtue of its oxygen to all fermentation—to all decomposition.

Do you believe that the tissues will remain indifferent to the presence of these putrid products? No. The dead nature in chemical reaction will be at war with the living tissues. It has been counselled, the tumor being open, to exercise no pressure upon it, to leave it to empty itself by the organic retraction of the tissues.

It is an error to believe as many do, that the air may enter every open cavity. The air by no means enters with facility the cavities whose walls are soft; the atmospheric column may often approach and apply these walls one against the other, instead of being introduced between them. But if the walls are inextensible, the atmosphere is incapable, by its weight, of effecting the occlusion of the cavity by the mechanism that I have just mentioned.

In our patient, the fear of the introduction of the air into the cavity is well founded; the central parietes as well as the parietes of the liver are but slightly flexible; this inevitable introduction will not, however, produce a direct excitation of the tissues, but will simply afford a chemical menstruum to the morbid liquids which they will secrete. In what manner shall we oppose the decomposition? In these cavities, it is the practice to make detergent antiseptic injections, that are made to lodge there for a certain length of time by closing the opening, which may be done by various means.

Some days after, the inflammation assumed a bad character, pus was effused, and, escaping into the abdomen, a peritonitis was developed, from the effects of which the patient died.

*Continuation and conclusion of M. Hugier's lectures on Catarrh of the Uterus. Diagnosis.*—We have already seen what are the characters proper to the discharges in catarrh of the uterus, but we should not, although these afford the principal and essential elements of the diagnosis, omit to inquire whether the disease be simple; whether due to a local or general cause; whether dependent or not on an affection of the tunic, of the neck, or of the body of the uterus; whether it is the consequence either of a lymphatic temperament, an anemic, or a scrofulous taint. These, you will at once perceive, are all very useful in furnishing the essential therapeutic indications, which we now proceed to speak of.

*Treatment.*—Our anato mico-pathological researches on catarrh of the uterus, rather than any purely therapeutical or speculative views, have led us to adopt a new medication which alone often proves efficacious after all the ordinary therapeutic means have failed. But we must repeat that it is important, nay, we should say indispensable, to examine into the causes to which the disease is owing—whether it be produced by excessive coition, onanism, syphilitic virus, etc. These circumstances, in fact, being known, certain therapeutical indications naturally present themselves to the mind. When the affection assumes an acute form and is accompanied by considerable reaction, local and general antiphlogistics are at once suggested; but it is not with this form that we usually have to do. It is rather with the chronic, and in such cases local antiphlogistics from the commencement are indicated.

If the catarrh is of an atonic nature, general and local tonic medicaments are to be insisted on. Along with these allow substantial and invigorating food; make use of aromatic and sulphur baths, seabathing; conjointly with these, free exercise in the open air, change of scene, moral stimuli, and the hundred *et ceteras* that will suggest themselves to the mind of the intelligent physician, may be made of very great assistance in the cure of his patient. Locally, tonic and astringent injections, with oak bark, quinine, rhatany, alum, etc., etc.

It has been thought, when these different means have failed to produce the desired result, that it would be useful to throw medicated injections into the cavity of the body of the uterus itself. This method, by no means new, was formerly somewhat in vogue, but had fallen entirely into oblivion, when M. Vidal (de Cassis) again endeavored to bring it into practice. It does not appear to have answered the expectations of practitioners, as indeed was clearly foreseen by those who possessed correct and precise ideas relative to the seat of the disease against which these means were directed. If you remember what we have said in relation to this subject, you will have no great difficulty in understanding why injections into the cavity of the body of the uterus can be of no avail. The explanation is found in the fact of the seat of the disease being in the neck and not in the body; and that its anatomical element, the follicular apparatus, being more or less profoundly seated in the tissue of the organ, and therefore but slightly accessible to the liquid injected. It is necessary then, if we wish to arrive at more certain results, that our remedies should be directed upon the seat of the disease. Moreover, we declare that, in our judgment, intra-uterine injections ought to be avoided, not so much because they are capable, according to the testimony of some physicians, of producing serious accidents, but rather because they do not attack the true seat of the disease, this not being connected with the internal membrane of the uterus, and are therefore inefficacious. One can easily conceive the inconveniences to which we have alluded. It is no hard matter to understand that stimulating caustic liquids thrown into the uterine cavity may traverse this organ, and pass through the Fallopian tubes into the peritoneum, and there produce the most serious accidents.

M. Vidal (de Cassis) admitted, it is true, the possibility of the dreaded consequences ensuing, which we have hinted at, but contends still for the advantages of intra-uterine injections, and lays the inconvenience and danger which may arise solely to the improper manner in which the application is made. Our *confrere*, however, has modified the mode of injection pursued in the commencement of this practice, and insists that the injection should be thrown up in a slow and gradual manner; that one should not only not use a tube whose calibre entirely fills up the neck, but that on the contrary, it should be free in order to facilitate the escape outwards of the injected liquid. If these rules are observed the injection cannot traverse the uterus and its tubes, and thus pass into the peritoneal cavity. We will even add that modified and administered as our colleague advises, intra-uterine injections will never be attended by any serious consequences. But it is not from fear of these accidents that we reject the method, but rather from a conviction that it is inoperative in a majority of cases; for not reaching the seat of the disease it can do good only in an indirect manner and in exceptional instances. Experience has taught us the insufficiency of the means proposed by Vidal, and it has done so by directing us to the anatomical element of the disease.

We will suppose that you have a case of uterine catarrh, and that, after having fulfilled the particular indications, examined the etiology and all the phenomena; in a word, that having taken into consideration everything proper to the disease, and used the ordinary list of remedies, the condition of your patient is in no degree amended—what would be the treatment? Our surgical therapeutics, and we wish here to speak only of them, would be as follows: Cauterizations, either with the nitrate of silver or hot iron; incisions, practiced in the cavity itself of the neck of the uterus, in order to destroy the mucous follicles which are the seat of the disease. These means may be used singly or associated together.

Let us now enter into some details on the subject. Cauterization with nitrate of silver is done by passing it over the whole surface of the neck, and all the part which projects into the vagina, when the follicles are the seat of the disease. To do this, you merely use a case armed with caustic of sufficient length to enable you to reach the neck—here you allow your caustic to remain one or two minutes; this being done, you withdraw the substance, and the operation is finished. Practiced in this way, cauterization has never produced serious accidents, either local or general, and the results have sometimes been satisfactory, or in other words efficacious, though it has very often proved insufficient. If the affection were confined to the superficial follicles the cauterization would be all that was required, and there would be a strong chance, in the majority of cases, of effecting a cure. But if the disease be situated in the follicles lying at a greater or less depth in the substance of the tissue, it is easy to see that the cauterization would be insufficient. It follows, then, that in this case you must go deeper, and it is precisely with this view that we resort to incisions, which reach and destroy the seat of the disease.

When all other means have failed, we resort to incisions. For this purpose the speculum being introduced, we pass into the uterine cavity a bistoury with a truncated blade, narrow and bent. We then make eight or ten incisions, and sometimes more, into the tissue of the neck, the whole length of its internal surface. The instrument acts in pressing from behind forwards, as if we intended each time to withdraw it. These numerous longitudinal incisions should penetrate to the depth of a line, or even two, into the substance of the tissues, so as to arrive at the seat of the disease, and attain and destroy the follicles themselves. The incisions being made, withdraw the bistoury; and we are most generally in the habit of immediately afterwards cauterizing boldly and freely the internal surface of the neck, with a view of cauterizing to the very bottom of the fissures made by the bistoury. This operation, which from the manner in which it is begun appears so formidable and fearful, is usually borne without complaint of much pain. We have even seen women who seemed hardly aware of the operation, or who have only complained of a slight pricking sensation. We have never seen any serious accidents result from the operation. During the first twenty-four hours after it has been performed some women complain of slight pain above the pubis, which is sometimes attended with a slight febrile action; but very often it is not followed by fever at all, and as soon as this transient disturbance has [illegible]

ed there is always the most perfect calm. We have never seen metritis, peritonitis, or any important lesions of other more distant organs, produced by this operation.

It sometimes happens that there is a discharge of blood the day of the operation, but this is very rarely abundant, though we have seen instances of a true hemorrhage, resulting as was afterwards discovered, from the woman being either in, near, or just through her menstrual period. This you will see is a circumstance calling for the postponement of the operation.

Since we saw the cases to which we have just alluded, our practice is to operate during the intervals of the catamenial flux, and since we have adopted this course, we have not seen a repetition of the hemorrhage. We must add, however, that when we wish an abundant local sanguineous discharge, we must not only not avoid but absolutely take advantage of the menstrual period. Except under circumstances of this kind, it is the better practice to wait till some days after this has passed before having recourse to the operation.

This would be the proper moment to say a few words on the application of this method of uterine incisions to certain obstinate cases of amenorrhœa. Suffice it to say, however, that we have used it several times with success; that it should never be resorted to till all other means have failed; that we have never seen it followed by any serious local or general accidents; and that we shall endeavor to give the question all due attention at some future day; at which period we trust we shall establish that the employment of this means is much more natural and legitimate than would appear at first sight. Moreover, you will have an opportunity of judging of its efficacy and seeing its mode of application this morning, in the case of a woman suffering from an amenorrhœa which has proved rebellious to all the ordinary remedies.

Let us not terminate this lecture without recalling to your minds, that catarrh of the uterus may be symptomatic of different affections either of the neck or the body of the organ, and that these last affections being susceptible of cure, by ordinary means, the catarrh, as a consequence, in its time disappears. But this does not always happen, for the original disease may disappear and the catarrh persist, as if it were the primitive affection. In cases of this description it becomes necessary to have recourse to the means of which we have spoken, and which differ, as is seen, from those employed in ordinary dischaiges, of a nature different from that which makes uterine catarrh.

We have as yet only hinted at a third method of cauterization which may be of use in the treatment of the affection under consideration; viz: the application of the hot iron. This means can be substituted for the cauterization by nitrate of silver; in fact it should be preferred in cases where the mucous membrane of the uterus offers granulations and fungous growths, which the red iron either destroys or advantageously modifies. But what we have said of the insufficiency of nitrate of silver may be applied also to the red iron, viz: that cauterization alone very rarely effects a radical cure when the disease is seated in the cavity of the neck.

It is now some years since we have resorted to the surgical means of which we have spoken in rebellious forms of uterine catarrh, both in our hospital and private practice, where the efficacy has been witnessed by great numbers of pupils and many of our colleagues, and we can now affirm to you that they are free from danger to the patient from beginning to end, and their employment is nearly always followed by the most satisfactory results.

The details which we have gone into concerning the pathological anatomy of the disease have, we hope, sufficed to convince you that this method is entirely natural, and its results consequently less surprising and inexplicable.

To conclude, our anatomico-pathological labors and clinical researches relative to catarrh of the uterus, have enabled us—1st, to establish the real seat of this affection, which we have shown to exist in the neck; 2d, to indicate the distinctive character of the discharge belonging to the disease; and 3d, and finally, to propose the most rational and most efficacious therapeutical means to be employed in the treatment of this disease.

The bistoury which M. Hugier is in the habit of using, in the operation of incising the inner surface of the neck of the uterus, is of a very simple description, being merely a narrow elbowed blade two lines in width, with a long handle, at and a little before its junction with which the edge of the blade is rounded, while the remaining portion presents a cutting edge, with a slightly blunted point. The only thing which he insisted on the student's remarking was, the very slight angle formed at the elbow or juncture of the two parts of the blade, by means of which form it accomodates itself to the parts and admits of greater facility of use than is offered by a straight bistoury.

For several days past I have been laboring under influenza —called here *la grippe*—which is prevailing in Paris at this time. Velpeau and several other members of the Faculty, from the very tight way in which it has held them, have been obliged to suspend their various hospital duties. Velpeau was so much indisposed that he did not make his usual visit this morning.

Paris, November 30, 1847.

